# Dual-Model Derailment Detection Algorithm Based on Variational Bayesian Kalman Filtering

**DOI:** 10.3390/mi15080939

**Published:** 2024-07-23

**Authors:** Shiwei Fan, Xu Gao, Ya Zhang, Huhe Chen, Guoxing Yi, Qiang Hao

**Affiliations:** 1 School of Instrumentation Science and Engineering, Harbin Institute of Technology, Harbin 150001, China; fanshiwei@hit.edu.cn (S.F.);; 2 School of Astronautics, Harbin Institute of Technology, Harbin 150001, China; 3 China Ship Research and Development Academy, Beijing 100101, China

**Keywords:** railway freight, derailment detection, MEMS IMU

## Abstract

A derailment detection algorithm for railway freight cars based on micro inertial measurement units was designed to address the complex issue of the disassembly and assembly of derailment braking devices. Firstly, a horizontal attitude measurement model for freight cars was established, and attitude measurement algorithms based on gyroscopes and accelerometers were introduced. Subsequently, a high-precision attitude measurement algorithm based on variational Bayesian Kalman filtering was proposed, which used acceleration information as the observation data to correct attitude errors. In order to improve the accuracy of derailment detection, a dual-model instantaneous attitude difference measurement technique was further proposed. In order to verify the effectiveness of the algorithm, offline data from simulation experiments and in-vehicle experiments were used to validate the proposed algorithm. The results showed that the proposed algorithm can effectively improve the measurement accuracy of horizontal attitude changes, reducing the error by 89% compared to pure inertial attitude calculation, laying a technical foundation for improving the accuracy of derailment detection.

## 1. Introduction

With the development of railway technology in China, the demand for freight trains is increasing day by day. As the service time of the railway line increases, the occurrence of long-wave irregularities in the track becomes increasingly common. Its characteristics are complex mechanisms and a wide range of wavelengths, such as simple supported beam deformation, roadbed settlement, bridge deflection deformation, etc. It may also exist at changes in the slope of the railway line, switch areas, road bridge junctions, etc. This situation increases the risk of the derailment of freight trains.

**Figure 1:**
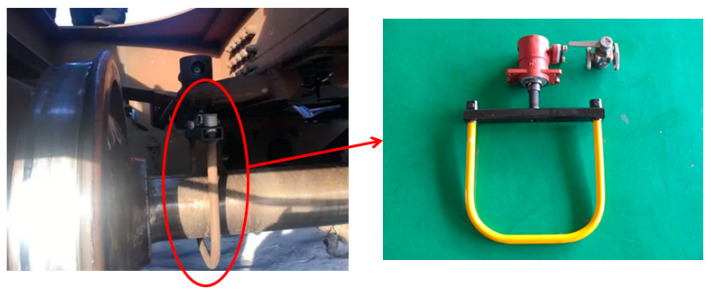
Freight train derailment detection and braking device.

At present, the derailment braking device of existing freight trains is mechanically operated, which collects derailment information by gripping the axle with a pull ring and top beam, as shown in Figure 1. Its function is reliable, but it requires a large amount of work to remove (install) the pull ring during vehicle maintenance and is an emergency response after vehicle derailment, and it does not have the function of preventing and controlling vehicle derailment in advance. The method proposed in the manuscript is to detect derailment by measuring the horizontal attitude angle. When the train is about to derail, the horizontal attitude changes. By collecting experimental data and setting reasonable thresholds, early braking can be applied when there is a significant change in horizontal attitude to prevent and control freight train derailment and reduce losses.

With the development of information technology and its deepening integration with traditional mechanical technology, the early prevention and control of vehicle derailment is becoming possible. In addition, the safety of railway transportation in the new era is receiving increasing attention. In order to further improve the safety of railway freight train operation, build a platform for the development of vehicle derailment early prevention and control technology, and resolve the concern of users to reduce the workload of lifting (dropping) trains, conducting research on the new generation of railway freight train derailment braking device technology based on information technology is of great significance for the safe operation of freight trains.

**Figure 2:**
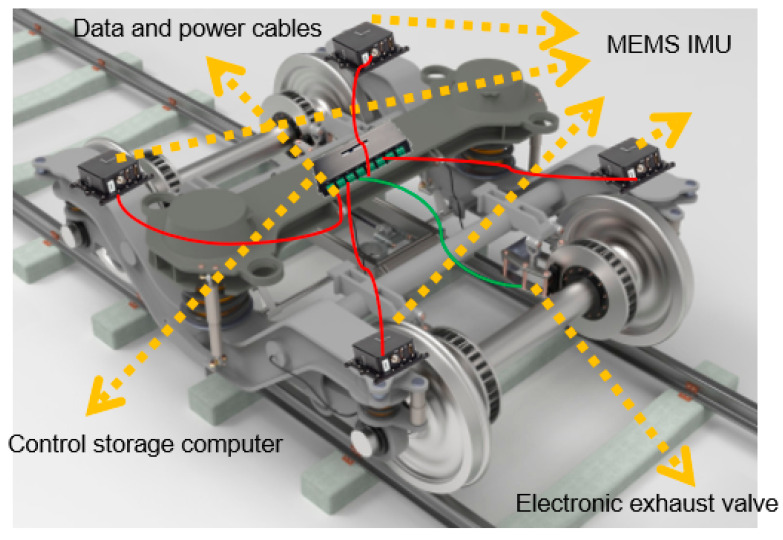
Diagram of derailment detection system components and installation.

We propose a new derailment detection device based on horizontal attitude, which installs four inertial measurement units, one control storage computer, one electrically controlled exhaust valve, and a battery on each freight train. The inertial measurement unit is installed on the train bogie (as shown in Figure 2), connected to the control storage computer through the CAN port, and sends measured acceleration and angular velocity information; we control the storage computer to receive acceleration and angular velocity information. Firstly, we use the initial alignment algorithm to obtain the initial pitch angle and roll angle. Then, combined with real-time data, we use the attitude update algorithm to calculate the horizontal attitude information online. Based on the preset threshold, we determine whether the current train is in a derailment state. If it is determined that it is about to derail, we send a command to the electric control exhaust valve. After receiving the command, the electric control exhaust valve performs train braking.

When a freight train derails, the wheels instantly land, causing a significant change in the horizontal attitude in a short period of time. The core of using information technology for derailment detection and braking lies in how to conduct a high-precision horizontal attitude measurement. Due to cost reasons, using high-precision inertial navigation systems to detect horizontal attitude information is unrealistic. The freight train targeted by our designed device does not have power in actual scenarios, and batteries need to be used to power the device. Therefore, the method in this paper mainly focuses on train derailment detection in low-cost and low-power scenarios. Therefore, it is of great engineering significance to utilize low-cost and high-precision MEMS inertial measurement units to achieve a high-precision horizontal attitude information measurement.

Due to the large bias of low-cost MEMS gyroscopes, attitude estimation cannot be provided separately. MEMS accelerometers are usually packaged together with gyroscopes to form inertial measurement units (IMUs). The three-axis accelerometer measures the gravitational force of acceleration, and the horizontal attitude can be determined by the component of the gravity vector on the horizontal axis. However, even under very stable conditions, there is still a lot of noise in the horizontal attitude measured by the accelerometer [1,2,3,4]. Therefore, complementary filters or Kalman filters are used to fuse these two sensors to overcome their respective shortcomings and obtain an overall accurate attitude estimation [5,6,7]. This fusion works well in static or quasi-static states because the accelerometer readings are dominated by gravitational acceleration, but under dynamic conditions, the accelerometer also measures linear or external acceleration, making its measurement results unsuitable for attitude determination. The compensation for this external acceleration must be conducted in some way to extract the gravity acceleration measurement and determine the attitude. Only in this way can it be integrated with gyroscope estimation. Another meaning of this issue is that it is not possible to use an accelerometer to measure the acceleration of a truck, as it also measures gravitational acceleration.

In order to accurately estimate attitude using MEMS, researchers have studied various adaptive algorithms using acceleration and magnetometer measurements. In the early stage, adaptation decisions were made by comparing the accelerometer norm, magnetometer norm, and tilt angle with their reference values [8]. Article [9] implements fuzzy rules for detecting acceleration and adjusting measurement covariance in an attitude reference frame. Some articles also propose the modeling of acceleration or magnetometer disturbances [10,11,12]. Suh proposed an adaptive rule that uses eigenvalues and vectors to calculate the correct innovation covariance using non-negative measurement covariance angles [13]. In terms of finding the fitness coefficient for measuring covariance, an algorithm for minimizing the Frobenius norm of innovative covariance was also proposed [14]. Li and Wang propose an adaptive algorithm that divides acceleration conditions into three stages: uniform motion, low-acceleration motion, and high-acceleration motion [15].

The current research has effectively improved the accuracy of attitude measurement, but the accuracy is still insufficient to meet the requirements for detecting vehicle derailment. Therefore, this article proposes a dual-model derailment detection algorithm based on variational Bayesian filtering. The variational Bayesian algorithm is used to approximate and infer the time-varying parameters of the Kalman filtering model. While recursively estimating the state, the adaptive filtering suppresses the influence of motion acceleration on attitude estimation, which can also track the changing noise variance and update the introduced degree of freedom parameters, thereby enhancing robustness. On this basis, a dual-model algorithm based on variational Bayesian Kalman filtering for attitude estimation and pure inertial attitude update is designed to address the characteristics of freight train derailment. The error of pure inertial attitude update is regularly corrected, and the attitude change of pure inertial solution in a short period of time is used to determine whether the freight train derails.

The main contributions of this article include the following: ① an attitude estimation algorithm based on a variational Bayesian Kalman filter using a variational Bayesian algorithm was designed to address the issue of the impact of freight train acceleration and deceleration on the accuracy of the horizontal attitude measurement; ② a dual-model relative horizontal attitude measurement derailment detection algorithm was designed for the special scenario of freight train derailment during the derailment cycle.

## 2. Related Work

### 2.1. Gyroscope-Based Attitude Update Algorithm

We choose the “E-N-U” geographic coordinate system as the navigation reference coordinate system for the derailment detection system, denoted as the *n* frame. Therefore, the attitude differential equation using the *n* frame as the reference system is

(1) $${\dot{C}}_{b}^{n}=C_{b}^{n}(\omega_{nb}^{b}\times )$$

where the matrix $C_{b}^{n}$ represents the attitude matrix of the body frame (*b* frame) relative to the *n* frame (which can be converted to attitude angle). Since the output of the gyroscope is the angular velocity of the b frame with respect to the inertial coordinate system (*i* frame), and the angular velocity information $\omega_{nb}^{b}$ cannot be obtained by direct measurement, it is necessary to transform differential Equation (1) as follows:

(2) $$\begin{array}{l} {\dot{C}}_{b}^{n}=C_{b}^{n}[(\omega_{ib}^{b}-\omega_{in}^{b})\times ]=C_{b}^{n}(\omega_{ib}^{b}\times )-C_{b}^{n}(\omega_{in}^{b}\times )\\ \;\;=C_{b}^{n}(\omega_{ib}^{b}\times )-(\omega_{in}^{n}\times )C_{b}^{n} \end{array}$$

where $\omega_{in}^{n}$ denotes the rotation of the navigational system with respect to the inertial frame, which consists of two parts, the rotation of the navigational system caused by the rotation of the Earth and the rotation of the *n* frame due to the curvature of the Earth’s surface as the system moves near the Earth’s surface, i.e., there is $\omega_{in}^{n}=\omega_{ie}^{n}+\omega_{en}^{n}$, where

(3) $$\omega_{ie}^{n}={\left[\begin{array}{ccc} 0 & \omega_{ie}\cos L & \omega_{ie}\sin L \end{array}\right]}^{T}$$

(4) $$\omega_{en}^{n}={\left[\begin{array}{ccc} -\frac{v_{N}}{R_{M}+h} & \frac{v_{E}}{R_{N}+h} & \frac{v_{E}}{R_{N}+h}\tan L \end{array}\right]}^{T}$$

where $\omega_{ie}$ is the angular rate of rotation of the Earth, and *L* and *h* are the geographic latitude and altitude, respectively.

The initial value of the attitude is obtained by the initial alignment algorithm, which is not described in this paper. When the attitude is calculated by Equation (2) with the initial alignment already in place, the error in angular velocity $\omega_{ib}^{b}$ measured by the low-cost MEMS gyroscope accumulates over time, resulting in a gradually larger attitude error.

### 2.2. Accelerometer-Assisted Horizontal Attitude Estimation Algorithms

In the absence of maneuvering, the input to the accelerometer in the navigational coordinate system is ${[0,0,-g]}^{T}$ (g is the acceleration due to gravity), then there are

(5) $$\left(\begin{array}{c} f_{x}^{b}\\ f_{y}^{b}\\ f_{z}^{b} \end{array}\right)=\left(\begin{array}{c} g\sin\theta \\ -g\cos\theta \sin\gamma \\ -g\cos\theta \cos\gamma \end{array}\right)$$

The pitch θ and roll γ angles can be obtained as

(6) $$\left\{\begin{array}{c} \theta =\arcsin\left(\frac{f_{x}^{b}}{g}\right)\\ \gamma =\arctan\left(\frac{f_{y}^{b}}{f_{z}^{b}}\right) \end{array}\right.$$

where ${\left[\begin{array}{ccc} f_{x}^{b} & f_{y}^{b} & f_{z}^{b} \end{array}\right]}^{T}$ is the measured value of the accelerometer in the *b* frame.

When the platform is maneuvering, the accelerometer measurements include not only the gravitational acceleration, and at this time, there is a large error in solving the attitude using Equation (6).

The attitude angle calculated by the accelerometer when the freight train is accelerating or decelerating does not truly reflect the attitude of the vehicle; the attitude angle solved by the gyroscope has high accuracy in the short term, but in the long term, due to the drift and integration operations, the calculated attitude angle generates cumulative errors. Therefore, it is necessary to fuse these two kinds of attitude angles to obtain an accurate attitude angle.

## 3. Derailment Detection Algorithm Based on Attitude Measurement

### 3.1. Variational Bayesian Algorithm

The variational Bayesian (VB) algorithm is an approximation algorithm used to find the joint a posteriori probability density, which utilizes known model information, observation information, and a priori information to obtain an approximate solution to the joint a posteriori probability density of the state vector and unknown parameters. The core idea is to approximate the true joint a posteriori probability density *p*(*X*, θ|Z) by the easy-to-find and relatively free-form joint probability density, where *X* denotes the unknown state vector, θ denotes the unknown parameter, and *Z* denotes the observation vector.

The joint state and unknown parameter inference problem based on the VB algorithm can be expressed as the following optimization problem:

(7) $$\left\{\begin{array}{c} \left\{q(X),q(\theta )\right\}=\arg\min KLD(q(X)q(\theta )||p(X,\theta |Z))\\ s.t.\int q(X)dX=1,\int q(\theta )d\theta =1 \end{array}\right.$$

(8) $$\begin{array}{l} KLD(q(X)q(\theta )||p(X,\theta |Z))\\ =\iint q(X)q(\theta )\log\frac{q(X)q(\theta )}{p(X,\theta |Z)}dXd\theta \end{array}$$

Since the true joint posterior probability density is unknown, it is not possible to solve the minimization problem in Equation (7) analytically, and it is necessary to introduce a negative free energy function, and the log-likelihood function can be computed as follows using Bayesian formulas:

(9) $$\begin{array}{l} \log p(Z)=\log\frac{p(X,\theta ,Z)}{p(X,\theta |Z)}\\ =\iint q(X)q(\theta )\log\frac{p(X,\theta ,Z)}{p(X,\theta |Z)}\\ =F(q(X),q(\theta ))+KLD(q(X)q(\theta )||p(X,\theta |Z)) \end{array}$$

where logp(Z) denotes the log-likelihood function and F(q(X),q(θ)) denotes the negative free energy function.

It is processed using Bayes’ theorem and the normalization property of density:

(10) $$\begin{array}{l} F(q(X),q(\theta ))=\iint q(X)q(\theta )\log\frac{p(X,\theta ,Z)}{q(X)q(\theta )}dXd\theta \\ =\iint q(X)q(\theta )\log p(X,\theta ,Z)dXd\theta \\ -\iint q(X)q(\theta )\log q(X)q(\theta )dXd\theta \\ =\iint q(X)q(\theta )\log p(X,\theta ,Z)dXd\theta \\ -\int q(X)\log q(X)dX-\int q(\theta )\log(q(\theta ))d\theta \end{array}$$

Since log *p* (*Z*) is a constant independent of *q*(*X*) and *q*(θ), the problem of minimizing the distance to KL can be transformed into a problem of maximizing F(q(X),q(θ)). The optimal solutions *q*(*X*) and *q*(*θ*) can be found by defining the logarithmic density functions $\log\tilde{p}(X)$ and $\log\tilde{q}(\theta )$.

$\log\tilde{p}(X)$ and $\log\tilde{q}(\theta )$ are defined as follows:

(11) $$\begin{array}{l} \log\tilde{p}(X)=\int q(\theta )\log p(X,\theta ,Z)d\theta \\ +c_{X},s.t.\int \tilde{p}(X)dX=1 \end{array}$$

(12) $$\begin{array}{l} \log\tilde{p}(\theta )=\int q(X)\log p(X,\theta ,Z)dX\\ +c_{\theta },s.t.\int \tilde{p}(\theta )d\theta =1 \end{array}$$

The optimal solutions *q*(*X*) and *q*(*θ*) satisfy the following equations:

(13) $$\begin{array}{l} \log q(\varphi )=E_{\Theta^{\left(-\varphi \right)}}[\log p(X,\theta ,Z)]\\ +c_{\varphi },s.t.\int q(\varphi )d\varphi =1\\ \Theta ≜\{X,\theta \} \end{array}$$

where Θ denotes the set consisting of state vectors and unknown parameters; φ∈Θ denotes any element in the set Θ; $\Theta^{\left(-\varphi \right)}$ denotes the element remaining in set Θ after removing element φ; q(φ) denotes the approximate posterior probability density of φ; and $E_{x}$ denotes the expectation operation with respect to the posterior density *q*(*x*) of random variable *x*.

Since the state vectors and the unknown parameters are coupled with each other, a non-moving point iteration is chosen to solve Equation (13) to obtain the local optimal solution of q(φ). The solution of Equation (13) is based on the state vectors and the unknown parameters.

### 3.2. Horizontal Attitude Estimation Algorithm Based on Variational Bayesian Kalman Filtering

#### 3.2.1. System Model

The discrete-time system for horizontal attitude estimation used in railroad freight train derailment detection is denoted as

(14) $$\begin{array}{c} x_{k+1}=F_{k}x_{k}+w_{k}\\ z_{k+1}=H_{k}x_{k+1}+v_{k+1} \end{array}$$

where *k* is the sampling time, $x_{k+1}\in {ℜ}^{n}$ is the state vector, and $F_{k}\in {ℜ}^{n\times n}$ is the state transfer matrix. $z_{k+1}\in {ℜ}^{m}$ is the measure vector. $H_{k+1}\in {ℜ}^{m\times n}$ is the measurement matrix, and n and m are the dimensions of the state vector and measurement vector, respectively. $w_{k}$ is the process noise and $v_{k+1}$ is the measurement noise. The process noise obeys a Gaussian distribution.

(15) $$p(w_{k})=N(w_{k};0^{n},Q_{k})$$

where *p*(·) denotes the probability density function and $N(w_{k};0^{n},Q_{k})$ denotes a Gaussian distribution with mean $0^{n}$ and covariance $Q_{k}$.

We choose the horizontal misalignment angle and gyro zero-bias error as state variables and the horizontal component of the acceleration information measured by the accelerometer in the *n* frame as the measure variable, so the following applies:

(16) $$\begin{array}{c} x_{k}={\left[\begin{array}{ccccc} \phi_{x} & \phi_{y} & \varepsilon_{x} & \varepsilon_{y} & \varepsilon_{z} \end{array}\right]}^{T}\\ z_{k}={\left[\begin{array}{cc} f_{x}^{n} & f_{y}^{n} \end{array}\right]}^{T} \end{array}$$

where $\phi_{x}$ denotes the eastward misalignment angle, $\phi_{y}$ denotes the northward misalignment angle, $\varepsilon_{x},\varepsilon_{y},\varepsilon_{z}$ denotes the gyro drift in the *x*, *y*, and *z* axes, and $f_{x}^{n},f_{y}^{n}$ denotes the eastward and northward acceleration information.

The state transfer matrix can be expressed as

(17) $$F_{k}=\left[\begin{array}{ccccc} 1 & 0 & & -C_{b}^{n}(1)*H_{n} & \\ 0 & 1 & & -C_{b}^{n}(2)*H_{n} & \\ 0 & 0 & 1 & 0 & 0\\ 0 & 0 & 0 & 1 & 0\\ 0 & 0 & 0 & 0 & 1 \end{array}\right]$$

where $C_{b}^{n}(1)$ denotes the first row of matrix $C_{b}^{n}$, $C_{b}^{n}(2)$ denotes the second row of matrix $C_{b}^{n}$, and *H_n_* denotes the sampling period.

The measurement matrix can be expressed as

(18) $$H_{k}=\left[\begin{array}{ccccc} 0 & -g & 0 & 0 & 0\\ g & 0 & 0 & 0 & 0 \end{array}\right]$$

where *g* denotes the acceleration of gravity.

#### 3.2.2. Variational Bayesian Kalman Filter

In the variational Bayesian Kalman filter (VBKF) algorithm, the mutually independent state variables $x_{k}$ and the measurement noise covariance $R_{k}$ are used as random variables, i.e., parameters to be estimated.

At the *k* − 1 observation moment, the prediction process can be defined as follows, assuming that the prior distribution of the joint probability density function of the state variable and the measurement noise covariance is the product of a Gaussian distribution and an inverse Wishart distribution:

(19) $$\begin{array}{c} p(x_{k},R_{k}|z_{1:k})=p(x_{k}|z_{1:k})p(R_{k}|z_{1:k})\\ =N(x_{k-1}|{\hat{x}}_{k|k-1},P_{k|k-1})\mathrm{IW}(R_{k}|{\hat{v}}_{k|k+1},{\hat{V}}_{k|k-1}) \end{array}$$

where the inverse Wishart distribution probability density function IW(·) is defined as

(20) $$\mathrm{IW}(R|v,V)=\frac{{\left|V\right|}^{v/2}}{2^{vd/2}\Gamma_{d}\left(\frac{v}{2}\right)}{\left|R\right|}^{-\left(v+d+1\right)/2}e^{-\frac{1}{2}\mathrm{tr}\left[VR^{-1}\right]}$$

where ***R*** is a symmetric positive definite random matrix; the distribution parameter ***v*** is the degrees of freedom parameter; ***V*** is a symmetric positive definite matrix; *d* is the dimension of ***R***; $\Gamma_{n}\left(\cdot \right)$ denotes the multivariate gamma function; and tr[·] is the trace of the sought matrix.

In the process of variational updating, the true posterior probability distribution $p(x_{k},R_{k}|z_{1:k})$ is approximated as the product of $q_{x}(x_{k})=N(x_{k},P_{k})$ obeying a Gaussian distribution and $q_{R}(R_{k})=\mathrm{IW}(R_{k}|v_{k},V_{k})$ obeying an inverse Wishart distribution according to the standard variational Bayesian algorithm, and the KL distance between the true distribution and the approximated distribution is computed iteratively to infer the optimal estimated value of the parameter to be evaluated, and the scatter function KL(·) is defined as

(21) $$\begin{array}{c} KL\left[q_{x}(x_{k})q_{R}(R_{k})||p(x_{k},R_{k}|z_{k})\right]=\\ \int q_{x}(x_{k})q_{R}(R_{k})\times \ln\frac{q_{x}(x_{k})q_{R}(R_{k})}{p(x_{k},R_{k}|z_{k})}dx_{k}dR_{k} \end{array}$$

An important derivation of the variational updating process is specified as

(22) $$\begin{array}{c} KL\left[q_{x}(x_{k})q_{R}(R_{k})||p(x_{k},R_{k}|z_{k})\right]=\\ \int q_{x}(x_{k})q_{R}(R_{k})\times \ln\frac{q_{x}(x_{k})q_{R}(R_{k})}{p(x_{k},R_{k}|z_{k})}dx_{k}dR_{k}\\ \ln q^{\left(i+1\right)}(R_{k})=C-0.5(m+{\hat{v}}_{k|k+1}+2)\ln\left|R_{k}\right|-\\ 0.5\mathrm{tr}((E^{\left(i\right)}\left[z_{k}-H_{k}x_{k}\right])) \end{array}$$

where *q*(*i*) (·) denotes the approximate probability distribution of *q*(·) at the *i*th iteration, *C* is a constant independent of the form of the distribution, and m is the dimension of the observed real matrix. Further, the expectation part of Equation (22) is defined as $T_{k}^{\left(i\right)}$ and expanded as

(23) $$\begin{array}{c} T_{k}^{\left(i\right)}=E^{\left(i\right)}\left[\left(z_{k}-H_{k}x_{k}\right){\left(z_{k}-H_{k}x_{k}\right)}^{T}\right]=\\ \left(z_{k}-H_{k}{\hat{x}}_{k}^{i}\right){\left(z_{k}-H_{k}{\hat{x}}_{k}^{i}\right)}^{T}+H_{k}P_{k}^{i}H_{k}^{T} \end{array}$$

It is easy to see that $q^{\left(i+1\right)}(R_{k})$ obeys a new form of inverse Wishart distribution, i.e., $q^{\left(i+1\right)}(R_{k})=\mathrm{IW}(R_{k}|{\hat{v}}_{k}^{\left(i+1\right)},{\hat{V}}_{k}^{\left(i+1\right)})$, with distribution parameters ${\hat{v}}_{k}^{\left(i+1\right)}$ and ${\hat{V}}_{k}^{\left(i+1\right)}$, respectively:

(24) $$\left\{\begin{array}{c} {\hat{v}}_{k}^{\left(i+1\right)}={\hat{v}}_{k|k-1}+1\\ {\hat{V}}_{k}^{\left(i+1\right)}={\hat{V}}_{k|k-1}+T_{k}^{i} \end{array}\right.$$

Similarly, the logarithmic expression of the approximate distribution of the state of the system takes the following form:

(25) $$\begin{array}{c} \ln q^{\left(i+1\right)}(x_{k})=-0.5{\left(z_{k}-H_{k}x_{k}\right)}^{T}E^{\left(i+1\right)}[R_{k}^{-1}](z_{k}-\\ H_{k}x_{k})-{0.5(x_{k}-{\hat{x}}_{k|k-1})}^{T}P_{k|k-1}^{-1}(x_{k}-{\hat{x}}_{k|k-1}))+C=\\ -0.5x_{k}^{T}\left[P_{k|k-1}^{-1}+H_{k}^{T}{\hat{R}}_{k}^{i+1}H_{k}\right]x_{k}+\\ x_{k}^{T}[P_{k|k-1}^{-1}{\hat{x}}_{k|k-1}+H_{k}^{T}{\hat{R}}_{k}^{i+1}z_{k}]+C \end{array}$$

where ${\hat{R}}_{k}^{i+1}=E^{\left(i+1\right)}{\left[R_{k}^{-1}\right]}^{-1}={\left(v_{k}^{i+1}-m-1\right)}^{-1}V_{k}^{i+1}$. Since *q*(***x***_k_) obeys a Gaussian distribution, i.e., $q(x_{k})=N(x_{k}|{\hat{x}}_{k},P_{k})$, the control gain $K_{k}^{i+1}$, the system state ${\hat{x}}_{k}^{i+1}$, and the state covariance $P_{k}^{i+1}$ in the variational measurement update are corrected as follows:

(26) $$\left\{\begin{array}{c} K_{k}^{i+1}=P_{k|k-1}H_{k}^{T}{\left(H_{k}P_{k|k-1}H_{k}^{T}+{\hat{R}}_{k}^{\left(i+1\right)}\right)}^{-1}\\ \begin{array}{c} {\hat{x}}_{k}^{i+1}={\hat{x}}_{k|k-1}+K_{k}^{i+1}(z_{k}-H_{k}{\hat{x}}_{k|k-1})\\ P_{k}^{i+1}=(\hat{x}-K_{k}^{i+1}H_{k})P_{k|k-1} \end{array} \end{array}\right.$$

From the above derivation and analysis, it can be seen that Equations (23), (24) and (26) constitute the implicit solution of the variational updating equation, and the optimal estimation of the parameter to be estimated at time k can be obtained by using the expectation–maximization algorithm for repeated iterative calculations.

### 3.3. Dual-Model Relative Horizontal Attitude Measurement Derailment Detection Algorithm

The height of the railroad track is generally less than 200 mm, and the length of each car in a railroad freight train is generally less than 25 m. The change in derailment time t and derailment pitch angle Δθ can be expressed as follows:

(27) $$\begin{array}{c} t=\sqrt{\frac{2h}{g}}\approx \sqrt{\frac{0.4}{9.8}}=0.202s\\ \Delta \theta =\frac{0.2}{25}=0.008\mathrm{rad}\approx 0.46^{{}^{\circ}} \end{array}$$

**Figure 3:**
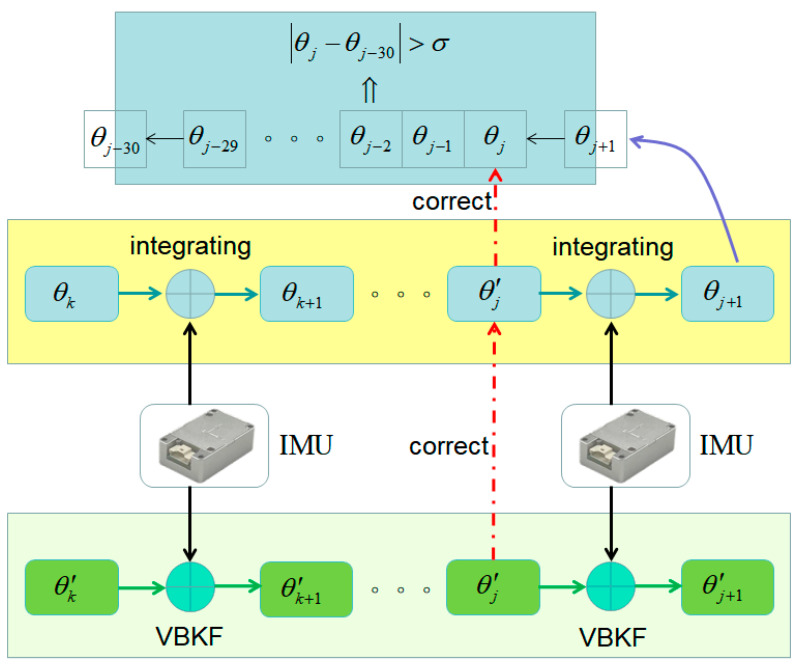
Dual-model derailment detection algorithm flow.

Therefore, the derailment time of the freight train is very short, and we can judge whether it is derailed or not by accurately measuring the attitude change within 0.3 s. Because the acceleration and deceleration process of the railroad freight train has a great influence on the horizontal component of the n-system horizontal acceleration, the filtering results have up and down fluctuations during acceleration and deceleration, and this fluctuation affects the judgment of train derailment. Therefore, this thesis proposes a dual-model derailment detection algorithm, using the gyroscope to calculate the attitude angle of a short period of time with high accuracy, so the use of this purely inertial attitude-solving algorithm is to calculate Δθ, to determine whether it is greater than the set threshold for derailment detection σ, and, through variational Bayesian Kalman filtering, to periodically correct the purely inertial solving results to protect its long-term measurement accuracy, and the specific process shown in Figure 3.

## 4. Experiments

**Figure 4:**
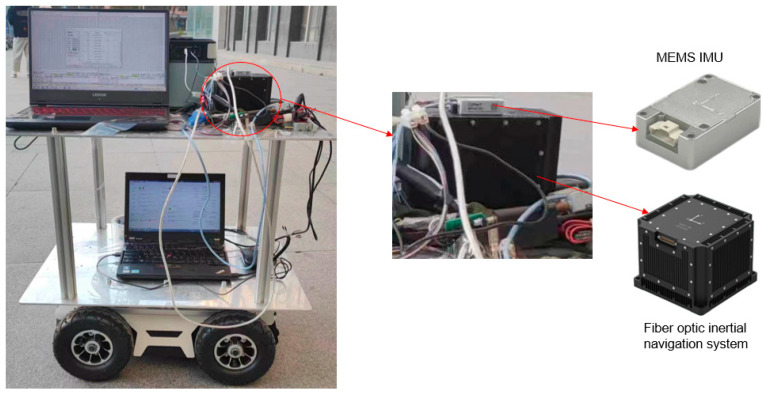
Diagram of the experimental equipment.

In order to verify the effectiveness of the algorithm proposed in this paper in processing real data, the on-board experimental data are utilized for offline processing and analysis. Due to the condition limitation, the on-board experiment utilizes an unmanned vehicle instead of a freight train and uses IMU with a gyro zero bias of 10°/h and an accelerometer zero bias of 0.2 mg for testing. The test uses a high-precision fiber optic gyro inertial navigation system as the attitude reference, and the equipment in the test is shown in Figure 4. The experimental trajectory includes turning, acceleration, deceleration and other scenarios, so we can verify the effectiveness of the method.

The experimental setup utilized MEMS gyroscope parameters as detailed in Table 1. The onboard vehicle experiment aimed to simulate the experimental conditions described in the third section. The unmanned vehicle traversed over stepped and bumpy road segments to simulate train derailment phenomena for experimental testing. Data collected from these experiments were processed accordingly.

**Figure 5:**
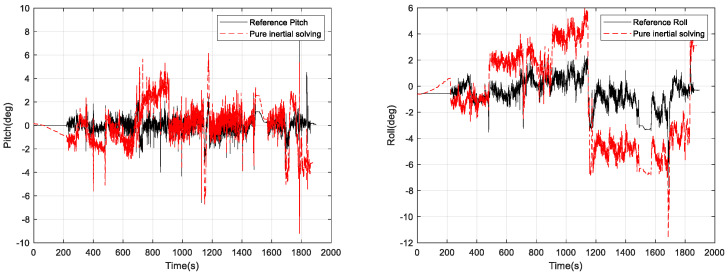
Pure inertial attitude solution results.

The experiment time is 1875 s. The results of pure inertial attitude solving relying only on the gyroscope are shown in Figure 5. The reference value in Figure 5 is the horizontal attitude information measured by the high-precision inertial navigation system. The horizontal attitude measurement error is less than 0.05 degrees, which can meet the requirements of the measurement process for the reference value.

**Figure 6:**
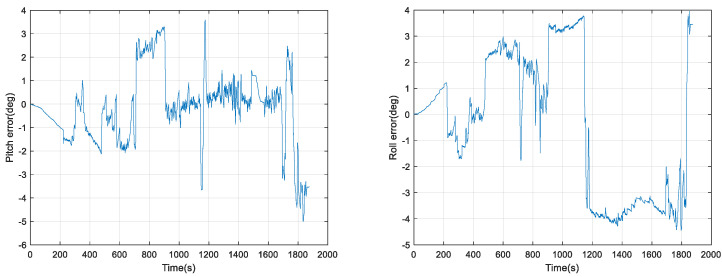
Pure inertial attitude solution error.

From Figure 5, it can be seen that the pure inertial attitude solution using gyroscopes is significantly different from the attitude reference, and further calculation of the error is shown in Figure 6.

From Figure 6, it can be seen that the pure inertial attitude solving error of the IMU in the test is very large, and the maximum error reaches −5°. In the first 200 s of which there is no movement of the cart, the error disperses with time, and later, due to the movement of the cart and the turn, the two horizontal misalignment angle errors are coupled to affect the attitude angle errors, resulting in the conclusion that the attitude angle error does not appear to exhibit a smooth change.

**Figure 7:**
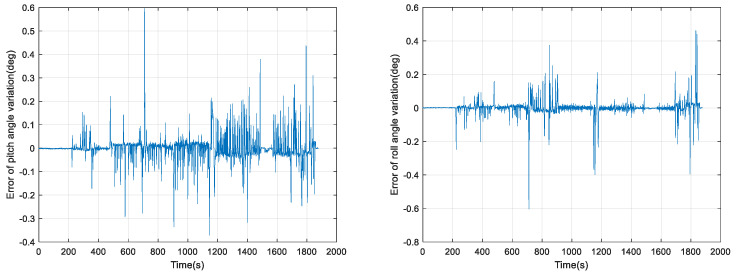
Error in the amount of horizontal attitude angle change in 0.3 s of purely inertial solution algorithm.

The error in the amount of horizontal attitude angle change for the purely inertial attitude solution in 0.3 s is shown in Figure 7.

**Figure 8:**
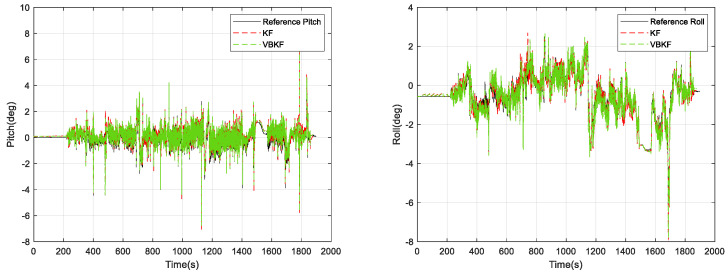
KF and VBKF algorithm attitude solution results.

**Figure 9:**
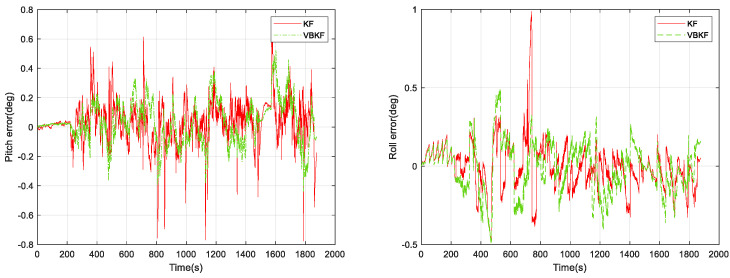
KF and VBKF algorithm attitude solution errors.

In Figure 7, the pure inertial attitude-solving algorithm reaches an error of 0.6° in the amount of pitch angle change and roll angle change, and the following results of our offline solving using Kalman filter (KF) and VBKF attitude estimation algorithms are shown in Figure 8 and Figure 9.

**Figure 10:**
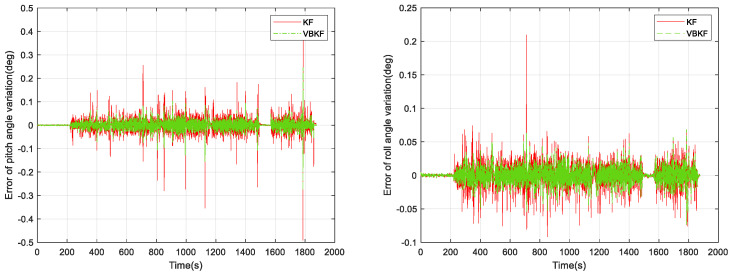
Error in the amount of horizontal attitude angle change in 0.3 s for KF and VBKF algorithms.

From Figure 9, it can be seen that the attitude solution using VBKF can significantly improve the attitude measurement accuracy compared with the pure inertial solution. Meanwhile, the pitch error of the KF attitude measurement algorithm is 0.776°, the roll error of the KF attitude measurement algorithm is 0.986°, the pitch error of the VBKF attitude measurement algorithm proposed in this paper is 0.521°, and the roll error of the VBKF attitude measurement algorithm proposed in this paper is 0.490°. The two algorithms whose pitch angle changes in 0.3 s compared with the reference frame are shown in Figure 10.

**Figure 11:**
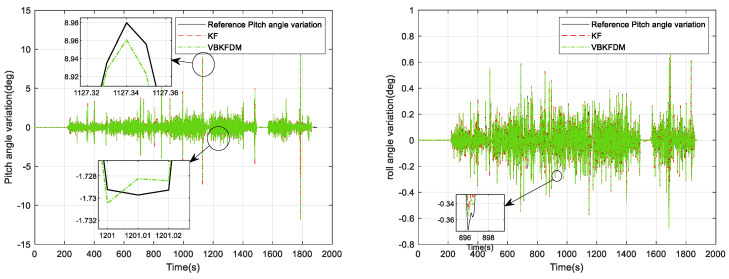
Amount of horizontal attitude angle change in 0.3 s for KF and VBKFDM algorithms.

**Figure 12:**
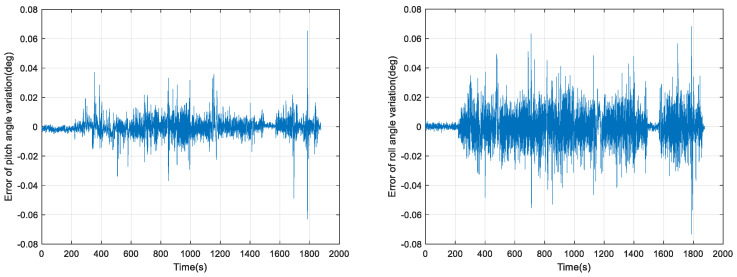
Error in the amount of horizontal attitude angle change in 0.3 s of VBKFDM algorithms.

From Figure 10, it can be seen that the errors of the KF attitude measurement algorithm in pitch angle change and roll angle change within 0.3 s are 0.491° and 0.209°, respectively. The errors of our proposed VBKF attitude measurement algorithm in pitch angle change and roll angle change within 0.3 s are 0.277° and 0.073°, respectively, which is a nonnegligible error for Δθ and can easily cause a misjudgment of the derailment detection threshold. We further validate the dual-model relative horizontal attitude measurement algorithm, and the results of the relative pitch angle change amount calculation within 0.3 s using the VBKFDM algorithm are shown in Figure 11 and Figure 12.

As can be seen in Figure 12, it can be seen that the pitch angle change error and roll angle change error within 0.3 s calculated by our proposed VBKFDM algorithm are 0.065° and 0.073°, respectively, which are much smaller than the horizontal attitude change when the train derails, and can meet the requirements of train derailment measurement.

## 5. Conclusions

This article studies a derailment detection method for railway freight trains based on micro inertial measurement units, designs a variational Bayesian Kalman filter (VBKF) attitude estimation algorithm, and proposes a pure inertial dual model coupled with VBKF to calculate the change in horizontal attitude angle during the derailment detection period. We finally validated the effectiveness of the algorithm proposed in this paper through in-vehicle testing. The maximum error of the pitch angle change calculated by the VBKFDM algorithm within 0.3 s is 0.073°, which is 85% smaller than the maximum error of the KF algorithm at 0.491°.

The algorithm proposed in this paper has wide applicability, and its measurement accuracy can meet the needs of derailment detection, which has important engineering significance.

## Figures and Tables

**Table 1:** Performance parameters of MEMS IMU.

	Parameter	Index
Gyro	Measuring range	±450°/s
	Bias stability	10°/s
	Scale factor nonlinearity	200 ppm
	Cross coupling	200 ppm
	Bandwidth	88.6 Hz@200 Hz
Accelerometer	Measuring range	±8 g
	Bias stability	0.2 mg
	Scale factor	200 ppm
	Cross coupling	200 ppm
	Vandwidth	88.6 Hz@200 Hz
Electrical characteristics	Voltage	5 ± 0.5 VDC
	Power consumption	0.3 W
Structural characteristics	Size	38 × 24 × 11.5 mm
	Weight	20 g
Usage environment	Operating temperature	−40 °C~+85 °C
	Storage temperature	−40 °C~+85 °C

## Data Availability

The original contributions presented in the study are included in the article, further inquiries can be directed to the corresponding author.
